# Lead Poisoning in Its Acute and Chronic Forms

**Published:** 1892-06

**Authors:** 


*Lead Poisoning in its Acute and Chronic Forms.* By THOMAS OLIVER, M.A., M.D., F.R.C.P. Pp. 121. Edinburgh: YOUNG J. PENTLAND. 1891.

This book consists of the Goulstonian lectures for 1891. The subject of plumbism is of great importance from its prevalence in the community, and it is, therefore, essential that every medical practitioner should be thoroughly acquainted with it. We think that in this work there will be found ample information on the many points of interest and practical importance which are connected with lead poisoning. The author has conducted experiments on the mode of absorption of lead by the digestive tract, and on the effects of the poison on animals. Illustrations of the microscopical appearances found in the organs affected are given in the form of beautiful coloured plates. There are also plates of the ophthalmoscopic changes in the optic disc and retina, and reproductions of sphygmograms. We would suggest that in future editions the nervous symptoms produced by the action of lead should be considered in greater detail, otherwise we have only praise for the clearness of the description and the lucid style in which the book is written.

